# Theoretical Biology and Medical Modelling: ensuring continued growth and future leadership

**DOI:** 10.1186/1742-4682-10-43

**Published:** 2013-07-11

**Authors:** Hiroshi Nishiura, Edward A Rietman, Rongling Wu

**Affiliations:** 1 Department of Global Health Policy, Graduate School of Medicine, The University of Tokyo, Hongo 7-3-1, Bunkyo-ku, Tokyo 113-0033, Japan; 2 Center of Cancer Systems Biology, GeneSys Research Institute, Tufts University School of Medicine, Boston 02142, USA; 3 Department of Statistics, Pennsylvania State University, University Park, PA 16802, USA; 4 Center for Statistical Genetics, Pennsylvania State University, Hershey, PA 10733, USA

## Abstract

Theoretical biology encompasses a broad range of biological disciplines ranging from mathematical biology and biomathematics to philosophy of biology. Adopting a broad definition of "biology", Theoretical Biology and Medical Modelling, an open access journal, considers original research studies that focus on theoretical ideas and models associated with developments in biology and medicine.

## Main text

*Theoretical Biology and Medical Modelling* (*TBioMed*), a 10 year old online journal, has steadily grown since its first launch in late 2003 as an independent journal of BioMed Central (BMC). Included in PubMed and PubMed Central and indexed by SCI for an impact factor, the journal has increasingly attracted a broad scientific audience, who are interested in mathematical modelling studies in biology and medicine [1]. We are pleased to announce that we have accepted the role of Editors-in-Chief of *TBioMed*, from June 2013, on behalf of all scholars in the community. We act as successors of Dr. Paul Agutter, a medical expert and mathematical modeller, who has made a respected effort to cultivate the field, grow *TBioMed* and let the journal be recognized across the world.

There have been four notable characteristics of *TBioMed* which we regard as advantageous for the authors and readers and we aim for these to remain unchanged. First, as an independent journal of BMC, *TBioMed* has continuously been an open access journal. The open access journal can permit all scientists across the world to have unrestricted and unlimited access to every single study in the field of theoretical biology and medicine. Instead of printing and selling the journal, the authors pay an article processing charge, thereby allowing all readers to download via internet and print the article at free of charge. Second, the online journal has another advantage for submitting authors, in that authors do not to have any limitation on the number of pages or figures and data that can be included, per article. For instance, the authors can decide whether a rigorous mathematical proof should be included in the main text or in the online only supporting material. Third, *TBioMed* makes a difference from other journals, in fulfilling the scope of the latter half of the title “Medical Modelling”, and attracting medical studies that are useful in applications to diagnosis, treatment and prevention. In fact, many published studies have been operational and directly applicable to existing medical problems. Fourth, the length of peer review is kept shorter than those of other journals in theoretical biology and medicine. We make an effort to ensure a fast review process, as we understand that operational studies cannot wait for months and years to reach publication, even when the study involves rigorous mathematical and analytical exercises.

In the field of theoretical biology and medicine, publications of new studies have tended to be restricted to those with an explicit methodological advancement and those significantly improving our understanding in biology. These types of publications, in particular, are seen in other good journals that have restricted scopes, such as *PLoS Computational Biology* or *Journal of Theoretical Biology*. Otherwise, original modelling studies are frequently considered by interdisciplinary journals, that remain to be rather broad and have very little restriction in the content (e.g. *PLoS ONE*). In relation to this point, we believe that *TBioMed* can beautifully fill its biomedical modelling niche, and indeed, the scope and standpoint of *TBioMed* are in line with this notion. We aim to offer a platform to publish, read and discuss relatively unrestricted studies, at the same time as having good quality control of the scientific content.

Under the Open Access publishing model, authors need to consider the financial support available to them to cover the article processing charge for publication in *TBioMed*[2]. For authors from developing nations in particular, with evident financial difficulty, BioMed Central operates an open access waiver fund [3] and authors who genuinely cannot afford to pay the article processing charge are able to request a discretionary waiver. Otherwise, authors are asked to promise to cover the charge upon submission, and in return, we promise to make an effort to justify the cost with academic merit. To justify the article processing charge, we aim to reach an improved impact factor and other citation metrics.

Presently, the impact factor of *TBioMed* is low to moderate, i.e., 1.46 in 2012, and other citation metrics are not substantially high (e.g. Eigenfactor is 0.00182). One of the most important roles for us during the next few years is to ensure greater impact and better metrics of the journal. Not only to recover and improve the impact factor, but we also aim to compete with and replace the aforementioned journals in the same subject category. We plan to promote special issues on topical subject areas and publish other special materials including award lectures. Moreover, we aim to contribute to education among students and early career researchers, and we will also consider a series of solicited review articles, written by invited leading scholars.

As we prepare to lead the journal, we would like to acknowledge the efforts that have been made by the founding editor, Dr. Denys Wheatley and former Editor-in-Chief, Dr. Paul Agutter in establishing the journal. Dr. Wheatley has worked on cell biology to better understand the mechanism of cancer, and most notably, he has been successful in raising and growing important journals in his professional areas including *Cancer Cell International*. Dr. Agutter has worked on a broad range of studies in theoretical biology, including the aetiology of deep venous thrombosis and chronic venous insufficiency, allometric scaling of metabolic rate, mechanisms of intracellular transport and history and philosophy of medicine and biology. We must emphasize that Dr. Agutter has spent a substantial fraction of his personal time in handling the manuscripts published in the journal to date, and we also thank him for his voluntary editing of submitted manuscripts written by authors in non-English speaking countries. Dr. Wheatley and Dr. Agutter are not only friends but also collaborators [4]. Dr. Ed Rietman, Professor Rongling Wu and I were put forward by Dr. Agutter as his successors, providing him with a chance to take a small step back from his busy life and benefit from having more personal time. As new Editors-in-Chief, all of us have contributed to the journal in different subjects [5-7], sharing different expertise and having experienced editorial jobs, as part of the editorial board of *TBioMed*. To deal with the broad range of subjects covered by the journal, new associate editors (who help manage peer-review) and editorial board members, who represent different subject areas, have been recruited. Moreover, we feel very confident in having Drs’ Wheatley and Agutter remain involved with the journal as associate editors. Splitting editorial responsibility into three, whilst supported by our strong colleagues on the board, we are taking over with a belief that *TBioMed* can be further strengthened and take the lead in its subject category.

We invite all authors in the community to consider *TBioMed* when submitting your original studies in the area of theoretical biology and medicine. We adopt a broad definition of "biology" and all original research studies that focus on theoretical ideas and models associated with developments in biology and medicine are considered. Submissions that are not only technically sound, but contributing to the field by offering either improved understanding in biology or progress in theory or methods will be highly welcomed.

## Competing interests

The authors declare that they have no competing interests.

## Authors’ contributions

This editorial was based on editorial meetings among all authors. HN drafted the manuscript. ER and RW gave comments on the earlier version of the manuscript. All authors read and approved the final manuscript.
